# Comparison of the 7th and revised 8th UICC editions (2020) for oral squamous cell carcinoma: How does the reclassification impact staging and survival?

**DOI:** 10.1007/s00428-023-03727-y

**Published:** 2024-01-08

**Authors:** Ann-Kristin Struckmeier, Philip Eichhorn, Abbas Agaimy, Mayte Buchbender, Tobias Moest, Rainer Lutz, Marco Kesting

**Affiliations:** 1 Department of Oral and Cranio-Maxillofacial Surgery, Friedrich-Alexander-Universität Erlangen-Nürnberg (FAU), Glückstraße 11, 91054 Erlangen, Germany; 2 Comprehensive Cancer Center Erlangen-European Metropolitan Area of Nuremberg (CCC ER-EMN), Erlangen, Germany; 3 Institute of Pathology, Friedrich-Alexander-Universität Erlangen-Nürnberg (FAU), Erlangen, Germany

**Keywords:** UICC, Staging, TNM classification, Oral squamous cell carcinoma

## Abstract

Since its introduction in 1968, the TNM (tumor, node, metastasis) classification established by the International Union Against Cancer has provided a consistent framework for staging of oral squamous cell carcinoma (OSCC). The introduction of the 8th edition in 2017 brought about significant modifications, encompassing the integration of depth of invasion (DOI) and extranodal extension (ENE) into the T and N classifications. Further, the UICC the criteria for the T3 and T4a categories were amended in 2020. This study aimed to evaluate the impact of reclassification on staging and, subsequently, the survival of patients with OSCC. Primary OSCCs from 391 patients were classified according to the 7th and revised 8th UICC editions (2020). Stage migration was assessed, and stage-specific progression-free survival (PFS) and overall survival (OS) were evaluated using the Kaplan–Meier method. The log-rank test was used to compare the different stages. Cox-proportional hazard modeling was used to compare the two editions. Incorporating the DOI into the T classification resulted in an upstaging of 77 patients, constituting 19.69% of the cohort. In addition, 49 (12.53%) patients experienced an upstaging when considering ENE in the N classification. Consequently, 103 patients underwent upstaging in UICC staging, accounting for 21.74% of cases. Upstaging mainly occurred from stage III to IVA (26.92%) and from stage IVA to IVB (31.78%). Upon comparing the categories in survival analysis, significant differences in OS and PFS were especially observed between stage IVB and lower stages. When examining the hazard ratios, it became evident that UICC 8 stage IVB is burdened by a 5.59-fold greater risk of disease progression than stage I. Furthermore, UICC 8 stage IVB exhibits a 3.83 times higher likelihood of death than stage I disease. We demonstrated significant stage migration from the 7th to the revised 8th UICC edition. Overall, incorporating DOI and ENE into the T and N classifications represents a substantial clinical advancement, leading to a more accurate staging of OSCC patients. Both staging systems exhibited statistically significant discrimination between stages; however, the 8th UICC edition allowed for a more precise categorization of patients based on their prognosis and led to enhanced hazard discrimination, particularly within higher stages.

**Supplementary Information:**

The online version contains supplementary material available at 10.1007/s00428-023-03727-y.

## Introduction

Oral squamous cell carcinoma (OSCC) accounts for approximately 90% of all malignant tumors originating in the oral cavity and is associated with a global annual incidence surpassing 350,000 cases [1, 2].

In order to make decisions regarding the optimal treatment strategies and estimate prognosis, the staging of OSCC patients is imperative.

A system delineating tumor extent, nodal spread, and distant metastasis of solid malignancies using the abbreviations “T” (tumor), “N” (node), and “M” (metastasis) was developed by Pierre Denoix in the 1940s [3]. In 1968, the International Union Against Cancer (Union internationale contre le cancer, UICC) introduced the first international classification of malignant diseases based on TNM [4]. Following, in 1977, the American Joint Committee on Cancer (AJCC) published its first staging manual [5]. The UICC and the AJCC staging manuals were subsequently aligned with each other, and they have since become indispensable in contemporary medicine for categorizing patients into staging groups, facilitating therapy planning, anticipating treatment outcomes, and thereby, estimating prognosis.

The foundational structure of the TNM classification remained the same until the 8th UICC edition, introduced in 2017, embraced a transformative approach by incorporating novel elements, namely depth of invasion (DOI) and extranodal extension (ENE), into the T and N classifications [6–8].

Previous editions encompassed criteria such as tumor size and infiltration of adjacent structures for T classification, and size and localization of lymph node metastases for N classification.

Nonetheless, numerous studies have highlighted the pivotal role of the DOI in OSCC [9–13]. As a result, DOI, defined as the distance from the basement membrane of the adjacent healthy squamous mucosa to the deepest point of invasion, was integrated into the UICC staging system.

Since 2017, OSCCs, previously classified as T1 in the 7th edition, are upstaged to T2 if they exhibit a DOI of 6–10 mm. Primary tumors formerly staged as T2, or in some cases T1, are reclassified as T3 when their DOI exceeds 10 mm. Furthermore, infiltration of the extrinsic tongue muscles was excluded from the T4a category of the 8th UICC edition [8].

However, in October 2020, the UICC issued an erratum, revising the definitions of the categories T3 and T4a [14]. In contrast to the previous criteria, a tumor greater than 4 cm in its largest dimension with a DOI exceeding 10 mm is now classified as T4a (previously T3). Tumors invading through the cortical bone of the mandible or maxilla, involving the maxillary sinus, or invading the skin of the face are still classified as T4a [14].

The presence of lymph node metastasis is known to significantly impact the prognosis of patients with OSCC [15]. However, a growing body of evidence in the literature underscores the additional impact of ENE on unfavorable outcomes [16, 17]. ENE is defined as the dissemination of tumor cells beyond the fibrous capsule into the adjacent tissue [18] and serves as a pivotal factor in the risk stratification of OSCC patients, including considering adjuvant therapy [19]. As a result, ENE was integrated into the 8th UICC edition [8].

Prior investigations on the comparison of the 7th and 8th UICC editions (2017) emphasized the dependence of stage migration on patient demographics and thereby, tumor localization [20].

Data on the revised 8th edition, published in 2020, are scarce. Hence, additional research on diverse patient populations is also needed for the 8th UICC edition (2020) to ensure global applicability and reliability.

This study sought to examine how adopting the revised 8th edition of the UICC classification, published in 2020, impacts staging and, consequently, survival of patients with OSCC.

Our study focused on evaluating changes in patients treated at a German high-volume medical center in accordance with the prevailing German guideline.

## Methods

### Patient cohort

The cohort consisted of patients with primary OSCC treated with radical tumor resection and neck dissection (ND; at least ipsilateral, supraomohyoid ND) according to the prevailing German guideline in a German high-volume center between January 1, 2015, and December 31, 2022.

Patients with recurrent OSCC or squamous cell carcinoma of the lip and those who did not undergo ND or ND with a decreased extent due to severe comorbidities were excluded.

Furthermore, we excluded patients who died within 30 days following surgery (perioperative death) from survival analyzes to avoid bias by surgery-related short-term mortality. In addition, patients with a follow-up of < 30 days were excluded.

The Ethics Committee of the Friedrich-Alexander-University Erlangen-Nuremberg approved the study’s design and methods (Ethic votes: 23–185-Br, 23–186-Br). In accordance with national regulations and institutional regulations, written informed consent was not required from the participating patients.

### Staging

In this study, all tumors were staged according to both the UICC 7 and UICC 8 (2020) criteria.

Tumor characteristics were assembled from hospital medical records and subsequently completed and validated by pathology files.

Regarding T classification, DOI was measured from the basement membrane of the adjacent healthy squamous mucosa to the deepest point of invasion.

Regarding N classification, ENE was defined as the dissemination of tumor cells beyond the fibrous capsule into the adjacent tissue.

### Clinicopathological characteristics

Clinicopathological characteristics were obtained from the clinical hospital files. The following parameters were systematically recorded and evaluated: age, sex, tumor localization, TNM classification, UICC stage, tumor size, DOI, histological grading, presence of perineural,, vascular, or lymphatic invasion, and ENE.

Progression-free survival (PFS) was defined as the time elapsed from the day of resection to locoregional or cervical/distant metastatic recurrence. Overall survival (OS) was defined as the time from the day of resection to death from any cause.

### Statistical analysis

Statistical analysis was performed using the Statistical Package for the Social Sciences 27.0 (SPSS, Chicago, IL, USA). Our assessment included evaluating stage migration, which is presented as both absolute numbers and percentages. In addition, stage-specific PFS and OS were calculated using the Kaplan–Meier method. Furthermore, we utilized the log-rank test to compare survival outcomes across different stages. Cox-proportional hazard modeling was used to compare the two editions regarding prognostic significance.

Figures were also created using SPSS.

Generally, a p value < 0.05 was considered statistically significant.

## Results

The cohort comprised 391 patients. 12 patients were excluded from survival analysis because of perioperative death or follow-up < 1 month.

**Fig. 1:**
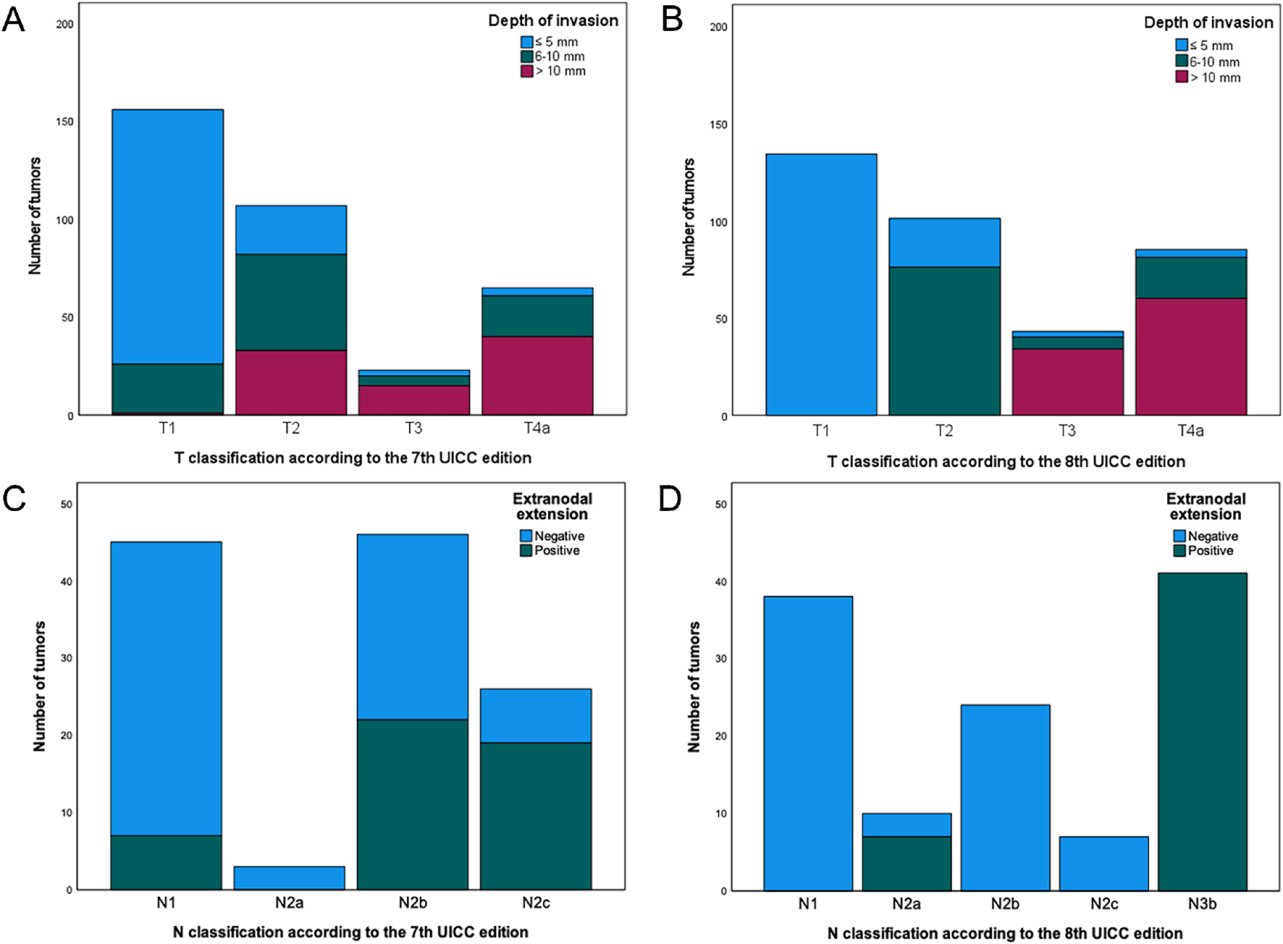
Categorization of tumors according to the T and N classifications of the 7th (A, C) and 8th UICC editions (B, D). In (A) and (B), depth of invasion is visually represented, whereas (C) and (D) depict the presence of extranodal extension in lymph node metastases

The included patients were predominantly male (238/391, 60.87%) with a median age of 64 (ranging from 31 to 92). The majority of the tumors were localized either at the floor of the mouth (132/391, 33.76%) or at the tongue (101/391, 25.83%). Cervical lymph node metastases were detected in 32.48% (127/391) of the patients. Among those patients, 77 (61.11%) exhibited ENE. DOI was ≤ 5 mm in 165 (42.20%) tumors, 6–10 mm in 103 (26.34%) tumors, and > 10 mm in 94 (24.04%) tumors. The complete clinical and demographic data are reported in Table 1. Figure 1 illustrates the distribution of DOI and ENE depending on the T and N classifications of the 7th and 8th UICC editions, respectively.

**Table 1:** Clinicopathological characteristics of the investigated cohort

Characteristics	Number of patients (%)
No. of patients	391
Sex	
Male	238 (60.87)
Female	153 (39.13)
Age	
Median	64
Range	31–92
Tumor localization	
Floor of the mouth	132 (33.76)
Tongue	101 (25.83)
Lower jaw	66 (16.88)
Upper jaw	38 (9.72)
Buccal plane	28 (7.16)
Palate	21 (5.37)
Multilocular	5 (1.28)
No. of resected LNs	
Median	35
Range	12–121
No. of resected metastatic LNs	
Median	0
Range	0–40
Histological grading	
G1	40 (10.23)
G2	199 (50.89)
G3	145 (37.08)
Gx	7 (1.79)
Lymphatic invasion	
L0	363 (92.84)
L1	27 (6.91)
Lx	1 (0.26)
Vascular invasion	
V0	384 (98.21)
V1	6 (1.53)
Vx	1 (0.26)
Perineural invasion	
Pn0	319 (81.59)
Pn1	6 (1.53)
Pnx	1 (0.26)
Residual tumor	
R0	381 (97.44)
R1	8 (2.0)
Rx	2 (0.51)
Depth of tumor invasion	
≤ 5 mm	165 (42.20)
6–10 mm	103 (26.34)
> 10 mm	94 (24.04)
DOIx	29 (7.42)
Extranodal extension (% of LNMs)	
ENE(-)	49 (38.89)
ENE( +)	77 (61.11)

Abbreviations: LN = lymph node, LNM = lymph node metastasis

Additionally, 59.59% (208/391) of the patients underwent adjuvant treatment, i.e., brachytherapy, radiation, or radiochemotherapy. 25 (6.39%) patients either declined adjuvant therapy or did not complete it, even though it was recommended.

### Shifts in T classification between the 7th and 8th UICC editions

In the 8th UICC edition, tumors were not only classified according to tumor size but also to their DOI. Tumors with a DOI between 6 and 10 mm are classified as T2, whereas tumors with a DOI > 10 mm are classified as T3.

Accordingly, 146 (84.88%) patients from the UICC 7 T1 group remained in the UICC 8 T1 group after reclassification, whereas 25 (14.53%) tumors with a DOI between 6 and 10 mm were reclassified as UICC 8 T2 and one tumor (0.58%) with a DOI > 10 mm was staged as UICC 8 T3.

Moreover, within the UICC 7 T2 category, 76 (68.47%) patients remained classified as UICC 8 T2. However, 35 (31.53%) tumors were reclassified as UICC 8 T3 due to their DOI surpassing 10 mm.

While the T3 category was severely underrepresented in the 7th UICC edition, growth was observed in the 8th UICC edition, published in 2017 (+ 9.20%). In contrast, with the erratum published in 2020, 16 tumors (64.00%) were reclassified as T4a tumors since their DOI exceeded 10 mm. As a result, 25 (6.39%) tumors were classified as T3 according to the 7th UICC edition, and 44 (11.25%) tumors were classified as T3 according to the 8th UICC edition.

Infiltration of the extrinsic tongue muscles was excluded from the T4a category of the 8th UICC edition. However, none of the tumors previously staged as T4a was downstaged. This could be attributed to the fact that, within our patient cohort, only one of the tumors localized at the tongue met the T4a classification criteria as defined by the 7th UICC edition.

In summary, 77 (19.69%) tumors were upstaged using UICC 8 criteria, and none of the tumors was downstaged.

Detailed data on the frequencies of the categories of the T classification according to the 7th and 8th UICC editions are provided in Table 2.

**Table 2:** Comparison between the 7th and 8th UICC editions

Characteristics	7th UICC edition (%)	8th UICC edition (%)
Pathological T classification		
T1	172 (43.99)	146 (37.34)
T2	111 (28.39)	102 (26.09)
T3	25 (6.39)	44 (11.25)
T4a	83 (21.23)	99 (25.32)
Pathological N classification		
N0	264 (67.52)	264 (67.52)
N1	50 (12.79)	42 (10.74)
N2a	3 (0.77)	11 (2.81)
N2b	47 (12.02)	24 (6.14)
N2c	27 (6.91)	9 (2.30)
N3b	0 (0.00)	41 (10.49)
UICC stage		
I	154 (39.39)	136 (34.78)
II	56 (14.32)	61 (15.60)
III	52 (13.30)	51 (13.04)
IV (7th edition)/ IVA (8th edition)	129 (32.99)	102 (26.09)
IVB		41 (10.49)

### Shifts in N classification between the 7th and 8th UICC editions

Overall, incorporating ENE into the N classification led to the upstaging of 49 (12.53%) out of 391 patients. There were no instances in which patients were downstaged using the criteria of the 8th UICC edition.

In the 7th UICC edition, 50 patients were classified as N1. Among this group, 8 (16.00%) patients were upstaged from UICC 7 N1 to UICC 8 N2a.

A total of 3 patients were classified as N2a (100%) in both UICC editions.

In the transition to the 8th UICC edition, N3 was further divided into N3a and N3b subcategories. Nevertheless, none of the patients were reclassified as N3a, as none of the metastases exceeded a size of 6 cm. However, a notable 24 (51.06%) patients underwent upstaging from UICC 7 N2b to UICC 8 N3b. Moreover, among the 27 patients classified as N2c according to UICC 7, 18 (66.67%) were reclassified as N3b according to the 8th UICC edition.

Detailed data on the frequencies of the N classification as per the 7th and 8th UICC editions can be found in Table 2.

### Shifts in tumor stage between the 7th and 8th UICC editions

Out of 150 tumors initially categorized as UICC 7 stage I, 17 (11.04%) underwent upstaging to UICC 8 stage II. This was primarily due to the transition of tumors from UICC 7 T1 to UICC 8 T2.

In addition, there was a single instance (0.65%) where a tumor initially staged as UICC 7 stage I was upstaged to UICC 8 stage III. This upstaging occurred due to the tumor's shift from T1 to T3, driven by its DOI exceeding 10 mm.

12 out of 56 (21.43%) patients were upstaged from UICC 7 stage II to UICC 8 stage III.

Furthermore, among the 52 tumors classified as UICC 7 stage III, 14 (26.92%) were reclassified as stage IVA according to UICC 8 criteria.

Moreover, among the 129 tumors initially staged as UICC 7 stage IVa, 41 (31.78%) were reclassified as UICC 8 IVB because of the presence of ENE.

In summary, 85 of 391 tumors (21.74%) experienced upstaging with the implementation of the 8th edition of UICC. There were no instances of tumors being downstaged using the updated UICC criteria.

Detailed data on the frequencies of the tumor stages according to the 7th and 8th UICC editions are provided in Table 2.

### Shifts in T and N classification depending on tumor localization

In the next step, we analyzed the shifts in T and N classification depending on tumor localization. Among the 132 tumors localized at the floor of the mouth, 25 (18.94%) were upstaged due to their DOI. Similarly, within the subset of tumors localized at the tongue, 26.73% (27/101) underwent upstaging in the T classification. Conversely, only 3.57% (1/27) tumors localized at the buccal plane were upstaged because of their DOI.

With regard to the N classification, an opposing pattern emerged. 40.82% (only patients with N + disease: 20/49) of the tumors localized at the floor of the mouth and 23.33% (only patients with N + disease: 7/30) of those localized at the tongue exhibited upstaging because of the presence of ENE. Conversely, in the case of tumors localized at the buccal plane, a substantial 57.14% (only patients with N + disease: 4/7) were upstaged.

Detailed data on the shifts in T and N classification depending on the tumor localization are provided in Table S1 and S2.

### Survival analysis and prognostic value of the 7th and 8th UICC editions

Survival data were available for 379 patients. 93 (24.5%) patients died during the follow-up period and 64 (16.9%) patients developed recurrences, i.e., local recurrence, cervical metastases, or distant metastases.

**Fig. 2:**
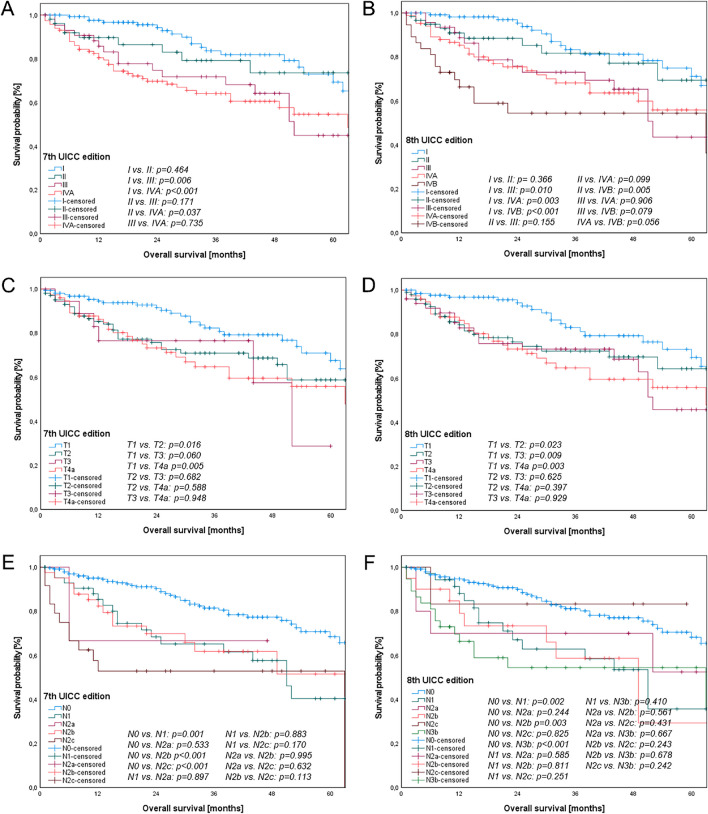
Kaplan–Meier curves of overall survival for OSCC patients according to T-, N- classifications and UICC stages of the 7th (A, C, E) and 8th UICC editions (B, D, F). (A) and (B) show significant discrimination between UICC stages according to both the 7th (p < 0.001) and 8th UICC editions (p < 0.001). In addition, (C) and (D) demonstrate significant differences between categories of the T classification of the 7th (p = 0.014) and 8th UICC editions (p = 0.010). (E) and (F) reveal significant distinctions between categories of the N classification of the 7th (p < 0.001) and 8th UICC editions (p < 0.001)

**Fig. 3:**
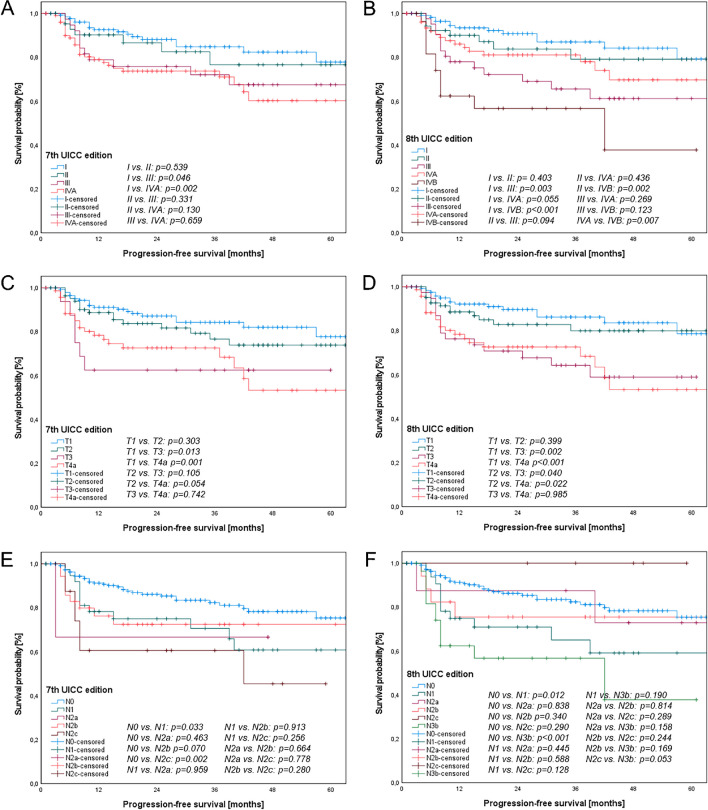
Kaplan–Meier curves of progression-free survival for OSCC patients according to T-, N- classifications and UICC stages of the 7th (A, C, E) and 8th UICC editions (B, D, F). (A) and (B) demonstrate significant differentiation between the UICC stages according to both the 7th (p = 0.014) and 8th UICC editions (p < 0.001) Furthermore, (C) and (D) reveal significant distinctions between categories of the T classification of the 7th (p = 0.005) and 8th UICC editions (p = 0.002). (E) and (F) indicate significant differences between categories of the N classifications of the 7th (p = 0.012) and 8th UICC editions (p < 0.001)

Overall, both staging systems showed statistically significant discrimination between stages for both OS (7th UICC edition: p < 0.001, 8th UICC edition: p < 0.001) and PFS (7th UICC edition: p = 0.014, 8th edition: p < 0.001) as shown in Figs. 2 and 3. Objective visual comparisons of the distribution and spread of survival curves between the competing UICC editions revealed more evenly spaced and monotonic curves for the 8th UICC edition, especially the T classification and UICC stages.

Figure 2a and b illustrate the OS according to the tumor stages of both UICC editions. Significant differences were observed between stage I and III and stage I and IVA in both editions (p < 0.05). Conversely, a significant difference was noted between stage II and IVA in the 7th UICC edition (p = 0.037), whereas this difference did not reach significance in the 8th UICC edition (p = 0.099). In addition, OS showed statistical significance between stage II and IVB in the 8th UICC edition (p = 0.005).

Figure 2c and d depict the OS curves according to the T classifications of the 7th and 8th UICC editions. Significant differences were evident between T1 and T2, as well as between T1 and T4a in both editions (p < 0.05). Additionally, a significant difference was observed between T1 and T3 in the 8th UICC edition (p = 0.009). However, the OS rates between T2 and T3, T2 and T4a, and T3 and T4a in both editions did not show significant differences (p > 0.05).

Figure 2e and f present the OS rates for the N classifications in both UICC editions. Significant differences were identified between N0 and N1, and N0 and N2b in both editions (p < 0.05). In the 7th edition, a significant difference was also observed between N0 and N2c (p < 0.001), whereas no statistically significant difference was found in the 8th UICC edition (p = 0.825). Conversely, a significant difference was evident between N0 and N3b in the 8th UICC edition (p < 0.001).

Figure 3a and b illustrate the PFS based on the tumor stages of both UICC editions. Significant differences were observed between stage I and III in both editions (p < 0.05). The difference between stage I and IVA reached significance in the 7th UICC edition but narrowly missed significance in the 8th UICC edition (p = 0.055). Notably, there was a statistically significant difference between stage I and IVB (p < 0.001), stage II and IVB (p = 0.002), and stage IVA and IVB (p = 0.007) in the 8th UICC edition.

Figure 3c and d illustrate the PFS according to the T classifications in both the 7th and 8th UICC editions. Significant differences were evident between T1 and T3, as well as T1 and T4a in both editions (p < 0.05). Furthermore, statistically significant differences were observed between T2 and T3 (p = 0.040) and T2 and T4a (p = 0.022) in the 8th UICC edition. However, both differences missed significance in the 7th UICC edition (p = 0.105 and p = 0.054, respectively).

Figure 3e and f present the PFS rates for the N classifications in both UICC editions. Significant differences were identified between N0 and N1 in both categories (p < 0.05). Furthermore, a significant difference was observed between N0 and N2c in the 7th UICC edition (p = 0.002). Conversely, a significant difference was evident between N0 and N3b in the 8th UICC edition (p < 0.001). The difference between N2c and N3b in the 8th UICC edition narrowly missed significance (p = 0.053).

### Comparison of the 7th and 8th UICC editions regarding hazard discrimination

Subsequently, Cox-proportional hazard modeling was employed. The findings revealed an increased likelihood of death and recurrence with a higher staging in both systems. Significant hazard discrimination was found between UICC 7 stage I and IVA (p = 0.003), whereas the difference between UICC 7 stage I and III narrowly missed significance (p = 0.054). Regarding the 8th UICC edition, significant hazard discrimination was found between stage I and IVA (p = 0.004) as well as stage IVB (p < 0.001). The difference between UICC 8 stage I and stage III narrowly missed significance (p = 0.052), similar to the 7th UICC edition.

When examining the hazard ratios, it became evident that UICC 8 stage IVB disease exhibited a 3.83 times higher likelihood of death than UICC 8 stage I disease. Moreover, regarding PFS, UICC 8 stage IVB exhibited a 5.60 times higher likelihood of disease progression than UICC 8 stage I disease. The detailed results of this analysis are presented in Table 3 and 4.

**Table 3:** Cox proportional hazard modeling for overall survival depending on 7th and 8th UICC edition

Stages	7th UICC edition			8th UICC edition		
	HR	95% CI	p value	HR	95% CI	p value
I	1 (Reference)			1 (Reference)		
II	1.26	0.61–2.63	0.535	1.16	0.55–2.45	0.706
III	2.39	1.30–4.39	0.005*	2.34	1.26–4.33	0.007*
IV/IVA	2.60	1.61–4.21	< 0.001*	2.24	1.39–3.85	0.004*
IVB				3.83	2.07–7.08	< 0.001*

Abbreviations: CI: confidence interval, HR: hazard ratio

A p value < 0.05 was considered statistically significant. Statistically significant differences are marked with an asterisk

**Table 4:** Cox proportional hazard modeling for progression-free survival depending on 7th and 8th UICC edition

Stages	7th UICC edition			8th UICC edition		
	HR	95% CI	p value	HR	95% CI	p value
I	1 (Reference)			1 (Reference)		
II	1.32	0.55–3.18	0.540	1.44	0.60–3.47	0.420
III	2.11	0.99–4.51	0.054	2.01	0.97–4.18	0.052
IV/IVA	2.50	1.37–4.58	0.003*	3.00	1.41–6.38	0.004*
IVB				5.60	2.54–12.31	< 0.001*

Abbreviations: CI: confidence interval, HR: hazard ratio

A p value < 0.05 was considered statistically significant. Statistically significant differences are marked with an asterisk

## Discussion

In order to make decisions regarding optimal treatment strategies and provide estimated prognoses, the process of staging is essential.

This study sought to examine how adopting of the revised 8th edition of the UICC classification, published in 2020, impacts staging and, consequently, survival of patients with OSCC. Our cohort included 391 OSCC patients treated at a German high-volume medical center in accordance with the prevailing German guideline.

During the 1980s, it became evident that tumor thickness plays a significant role in determining the prognosis of OSCC patients [21, 22]. However, in recent times, DOI has emerged as a more appropriate variable to consider [9–13]. DOI is deemed to be more suitable to identify locally invasive lesions and to distinguish them from superficial, exophytic growing tumors that exhibit a more indolent-acting behavior than tumor thickness [23].

Furthermore, a growing body of evidence in the literature underscores the substantial impact of ENE on the prognosis of OSCC patients [16, 17].

Among our study participants, the inclusion of DOI into T classification resulted in upstaging of 77 patients (19.69%). In contrast, the addition of ENE to the N classification resulted in upstaging of 49 patients (12.53%).

25 (14.53%) tumors were reclassified from UICC 7 T1 to UICC 8 T2, while 35 (31.53%) tumors showed upstaging from UICC 7 T2 to UICC 8 T3. Infiltration of the extrinsic tongue muscles was excluded from the T4a category of the 8th UICC edition. However, none of the tumors previously staged as T4a was downstaged. This could be attributed to the fact that, within our patient cohort, only one of the tumors localized at the tongue met the T4a classification criteria as defined by the 7th UICC edition.

Within the nodal staging, 8 of 50 patients (16.00%) were upstaged from UICC 7 N1 to UICC 8 N2a, and 24 of 47 tumors (51.06%) were upstaged from UICC 7 N2b to UICC 8 N3b due to the presence of ENE. Furthermore, 18 of 27 patients (66.67%) experienced upstaging from UICC 7 N3a to UICC 8 N3b.

Consequently, 103 out of 379 patients underwent upstaging in UICC stages, accounting for 21.74% of cases. Notably, upstaging mainly occurred from stage III to IVA (14/52, 26.92%) and from stage IVA to IVB (41/129, 31.78%).

Similar to our results, Matos et al. observed an upstaging rate of 22.8% in T classification in a cohort of 298 OSCC patients. However, they described a much higher upstaging rate in the N classification of 29.2% [24]. This might be due to the fact that they included a higher percentage of patients with advanced disease, which is unfortunately the reality in developing countries such as Brazil (UICC 7 N2c: 16.7% vs. 6.91% in our cohort).

Cramer et al. investigated migration in UICC stages in a cohort of 39,361 patients from the National Cancer Database and reported a surprisingly much lower upstaging rate of 10%, with the most notable increases occurring in stages UICC 7 II and III to UICC 8 IVB [25]. Conversely, Mate et al. observed an upstaging of 31% of their patients with the most migrations from UICC 7 stage IV to UICC 8 IVB. However, only 4.5% of their tumors were upstaged based on DOI, whereas 26.5% were upstaged based on the presence of ENE [26]. Nevertheless, it is important to emphasize that their patient cohort consisted of 57% tumors localized at the buccal plane.

Because of these results, we analyzed upstaging in T and N classification depending on tumor localization. In the group of tumors localized at the floor of the mouth, tongue, and buccal plane, 18.94%, 26.73%, and 3.57% were upstaged in T classification, respectively. When investigating the shifts in N classification, an opposing pattern emerged: 57.14% of the tumors localized at the buccal plane were upstaged, whereas only 40.82% and 23.33% of the tumors localized at the floor of the mouth and the tongue underwent upstaging.

These results are in accordance with those by Singhavi et al., who investigated a cohort of 863 patients from India and analyzed stage migration in patients with tumors of the tongue and buccal plane separately. They found OSCC localized at the tongue to have a higher stage migration in the early stages (I and II) as compared with those of the buccal plane. They attributed this to the increased DOI in tongue carcinomas. In contrast, they found OSCC localized at the buccal plane to have a higher migration in stage III due to an increased incidence of ENE [20].

Overall, in the Western world, where OSCC is predominantly localized at the tongue and the floor of the mouth, upstaging seems to be primarily driven by DOI. In contrast, within the Eastern world, dominated by OSCC localized at the buccal plane, the primary factor for upstaging appears to be the presence of ENE.

In the following step, we explored how the modifications to the staging criteria for T3 and T4 carcinomas, as outlined by the UICC in 2020 [14], influenced the classification of our patients.

In our patient cohort, 16 (4.09%) tumors, previously classified as T3, were upstaged according to the edition published in 2020 compared with the edition from 2017.

Hence, it is imperative to consider that previous research has investigated the changes in staging brought about by the transition to the 8th UICC edition, published in 2017. Data on the revised 8th edition, published in 2020, are scarce. Tagliabue et al. investigated the impact of the reclassification of OSCC localized at the tongue according to the 8th UICC edition (2020). Within their patient cohort, upstaging was observed in 9% of the patients, whereas 16% of the tumors experienced downstaging [27].

Cox-proportional hazard modeling revealed an increased likelihood of death and disease progression with higher overall staging in both systems. When examining the hazard ratios, it became evident that UICC 8 stage IVB disease exhibited a 3.83 times higher likelihood of death than UICC 8 stage I disease. According to the 8th UICC edition, UICC 8 stage IVB patients are burdened by a 5.60-fold greater risk of disease progression than UICC 8 stage I.

In contrast to our results, Tagliabue et al. reported a 4.84-fold elevated risk of death and a 3.13-fold higher risk of disease progression or the occurrence of secondary OSCC for patients in UICC 8 (2020) stage IV compared to patients in stage I [27]. Results regarding PFS might be attributed to the fact that they did not differentiate between UICC 8 stage IVA and IVB.

Upon survival analysis, the most significant alterations were observed in the advanced stages. In general, both staging systems exhibit statistically significant discrimination between stages for both OS and PFS; however, the 8th UICC edition, on the whole, allowed for a more precise categorization of patients based on their prognosis, particularly because of the introduction of the advanced stages N3b and IVB. This is in accordance with the results by Matos et al. [24], Kano et al. [28], and Singhavi et al. [20].

However, the current UICC edition is not without controversy.

Ebrahimi et al. demonstrated that tumors exhibiting bone invasion restricted to the cortical bone have a similar prognosis to those without bone invasion, suggesting that not all of the patients with bone invasion should be classified as T4a. Conversely, they recommended considering upstaging by one T category in cases of medullary bone invasion [29].

Barrett et al. advocated for removing of the invasion of extrinsic tongue muscles from the T4a category since the extrinsic muscles are of a relatively shallow nature and are sometimes affected by only superficial carcinomas [30]. In contrast, Liao et al. recommended retaining extrinsic muscle invasion as part of the classification for T4a tumors due to poorer outcomes compared with T3 tumors [31]. Nonetheless, in our study group, the involvement of extrinsic tongue muscles is of minimal significance. Only a single tumor localized at the tongue was classified as T4a according to the 7th UICC edition, and it retained this categorization after reclassification due to its DOI > 10 mm.

Furthermore, Subramaniam et al. suggested including histopathological features, such as perineural invasion, within the T classification [32].

In their study, Ho et al. explored the relationship between the number of lymph node metastases and survival rates. They observed a rising risk of mortality as the number of lymph node metastases increased, with the most significant impact seen when there were up to four lymph node metastases [33]. Ebrahimi et al. highlighted the significance of the number of lymph node metastases in OSCC as well, drawing from an international multicenter trial [34]. In 2017, the number of lymph node metastases was incorporated into the N classification for HPV-positive oropharyngeal squamous cell carcinoma, whereas this criterion was not applied to HPV-negative carcinomas. However, adding the number of lymph node metastases to the N classification might address the current limitations of the N classification of OSCC and should be considered in future research.

The prevailing UICC edition adheres to a rather traditional approach that relies on histopathological and anatomic information. However, an improved understanding of the molecular and genetic aspects of cancer has shifted the focus to precision oncology and personalized medicine.

In contemporary oncology, there is a growing emphasis on comprehending the influence of the tumor microenvironment on tumor progression [35]. The concept of an immune score, initially applied in colorectal cancer [36], is garnering growing interest due to its potential relevance in head and neck squamous cell carcinomas [37]. Zhou et al. devised an immunologically based prognostic scoring system that has demonstrated significant relevance in survival prediction [38]. Zhang et al. and Galon et al. classified tumors into two distinct categories: immune-hot (characterized by high numbers of CD3^+^ and CD8^+^ immune cells) and immune-cold (characterized by a low number of CD3^+^ and CD8^+^ immune cells) [39, 40]. While this assessment can assist in identifying cases that might benefit from immunotherapy, it also helps estimate the prognosis of OSCC patients [41].

Heikkinen et al. proposed an alternative strategy, advocating a comprehensive assessment of stromal tumor-infiltrating lymphocytes as a means of risk stratification in early-stage oral tongue squamous cell carcinoma [42].

Beyond the aforementioned criteria, several factors such as tumor budding [43–45], stromal reactions (including desmoplasia and local immune responses) [46], the tumor-stroma ratio [47, 48], the presence of myofibroblasts [49], and cancer-associated fibroblasts [50] may merit consideration for inclusion in the next generation of staging.

Our study has several limitations that should be considered. First, its retrospective and single-center design introduces inherent biases. Nevertheless, it is noteworthy that our study boasts a substantial sample size of 391 patients and a very homogenous patient cohort, distinguishing it from comparable studies.

Previous research indicates distinct patterns in upstaging upon tumor localization, as delineated previously. Therefore, we conducted analyzes on stage migration depending on tumor localization. However, our patient cohort only included a small number of tumors localized at the buccal plane (n = 28), reflecting the typical characteristics of the Western OSCC patients with a high prevalence of tumors localized at the tongue and floor of the mouth. The limited number of buccal OSCC in our study restricts its interpretative utility. However, our results emphasize the differences between OSCC localized at the tongue and buccal OSCC. Hence, additional research on diverse patient populations is required to ensure global applicability and reliability.

Nonetheless, it its worth emphasizing that we are the first to investigate the impact of reclassification by the revised 8th UICC edition, published in 2020, in a large cohort of OSCC patients with various tumor sites.

## Conclusion

In conclusion, we showed significant stage migration from the 7th to the 8th UICC edition. Upstaging was primarily driven by DOI in our patient cohort, presumably attributed to the fact that we investigated a Western cohort, dominated by OSCC localized at the tongue and floor of the mouth.

Overall, incorporating DOI and ENE into the T and N classifications represents a substantial clinical advancement, leading to a more accurate staging of OSCC patients. Both staging systems exhibited prognostic significance; however, the 8th UICC edition allowed for a more precise categorization of patients based on their prognosis, leading to enhanced hazard discrimination, particularly within higher stages.

The current UICC edition represents a significant step forward in capturing the complexities of the disease, but it adheres to a rather traditional approach. Including immunological and histopathological factors could enhance prediction accuracy and might allow a more precise categorization of patients, aiming toward a more personalized medicine. Future research should focus on the validation of these factors.

### Supplementary Information

Below is the link to the electronic supplementary material.

Supplementary file1 (DOCX 19 KB)

## Data Availability

The data that support the findings of this study are available from the corresponding author upon reasonable request.

## Abbreviations

AJCC: American Joint Committee on Cancer
DOI: Depth of invasion
ENE: Extranodal extension
N classification: Nodal classification
ND: Neck dissection
OS: Overall survival
OSCC: Oral squamous cell carcinoma
PFS: Progression-free survival
T classification: Tumor classification
TNM: Tumor, node, metastasis
UICC: Union internationale contre le cancer, International Union Against Cancer

## Declarations

This study was performed in line with the principles of the 1964 Helsinki Declaration and its later amendments. The Ethics Committee of the Friedrich-Alexander University Erlangen-Nuremberg approved the study’s design and methods (Ethic votes: 23–185-Br, 23–186-Br). In accordance with national regulations and institutional regulations, written informed consent was not required from the participating patients.
