# Supplementary material for: Comparison of the 7th and revised 8th UICC editions (2020) for oral squamous cell carcinoma: How does the reclassification impact staging and survival?
Source: Virchows Arch. 2024 Jan 8;484(6):901–13. doi: 10.1007/s00428-023-03727-y (PMC11186894; doi:10.1007/s00428-023-03727-y)
Supplement: Supplementary file 1 — Supplementary file1 (DOCX 19 KB) [file 428_2023_3727_MOESM1_ESM.docx]

**Supplementary Information:**

**Comparison of the 7th and revised 8th UICC editions (2020) for oral squamous cell carcinoma: How does the reclassification impact staging and survival?**

Ann-Kristin Struckmeier MD, DMD^1,2^*, Philip Eichhorn MD^2,3^, Abbas Agaimy MD^2,3^, Mayte Buchbender DMD^1,2^, Tobias Moest MD, DMD^1,2^, Rainer Lutz MD, DMD^1,2^, Marco Kesting MD, DMD^1,2^

^1^ Department of Oral and Cranio-Maxillofacial Surgery, Friedrich-Alexander-Universität Erlangen-Nürnberg (FAU), Glückstraße 11, 91054, Erlangen, Germany.

^2^ Comprehensive Cancer Center Erlangen-European Metropolitan Area of Nuremberg (CCC ER-EMN), Erlangen, Germany.

^3^ Institute of Pathology, Friedrich-Alexander-Universität Erlangen-Nürnberg (FAU), Germany.

* Corresponding author: Ann-Kristin Struckmeier, e-mail: ann-kristin.struckmeier@uk-erlangen.de, ORCID: 0000-0002-4015-1466

**Table S1** Comparison of the T classification of the 7th and 8th UICC editions depending on tumor localization

1. Floor of the mouth:

|  | | **8th UICC edition** | | | |
| --- | --- | --- | --- | --- | --- |
|  |  | **T1** | **T2** | **T3** | **T4a** |
| **7th UICC edition** | **T1** | 53 (84.13%) | **10 (15.87%)** |  |  |
|  | **T2** |  | 20 (57.14%) | **15 (42.85%)** |  |
|  | **T3** |  |  | 10 (100.00%) |  |
|  | **T4a** |  |  |  | 24 (100.00%) |

1. Tongue

|  | | **8th UICC edition** | | | |
| --- | --- | --- | --- | --- | --- |
|  |  | **T1** | **T2** | **T3** | **T4a** |
| **7th UICC edition** | **T1** | 43 (76.78%) | **12 (21.43%)** | **1 (1.79%)** |  |
|  | **T2** |  | 25 (64.10%) | **14 (35.90%)** |  |
|  | **T3** |  |  | 5 (100.00%) |  |
|  | **T4a** |  |  |  | 1 (100.00%) |

1. Lower jaw

|  | | **8th UICC edition** | | | |
| --- | --- | --- | --- | --- | --- |
|  |  | **T1** | **T2** | **T3** | **T4a** |
| **7th UICC edition** | **T1** | 14 (93.33%) | **1 (6.67%)** |  |  |
|  | **T2** |  | 13 (72.22%) | **5 (27.78%)** |  |
|  | **T3** |  |  | 4 (100.00%) |  |
|  | **T4a** |  |  |  | 29 (100.00%) |

1. Upper jaw

|  | | **8th UICC edition** | | | |
| --- | --- | --- | --- | --- | --- |
|  |  | **T1** | **T2** | **T3** | **T4a** |
| **7th UICC edition** | **T1** | 9 (100.00%) |  |  |  |
|  | **T2** |  | 4 (100.00%) |  |  |
|  | **T3** |  |  | 2 (100.00%) |  |
|  | **T4a** |  |  |  | 23 (100.00%) |

1. Buccal plane

|  | | **8th UICC edition** | | | |
| --- | --- | --- | --- | --- | --- |
|  |  | **T1** | **T2** | **T3** | **T4a** |
| **7th UICC edition** | **T1** | 16 (94.12%) | **1 (5.88%)** |  |  |
|  | **T2** |  | 7 (100.00%) |  |  |
|  | **T3** |  |  | 2 (100.00%) |  |
|  | **T4a** |  |  |  | 2 (100.00%) |

1. Palate

|  | | **8th UICC edition** | | | |
| --- | --- | --- | --- | --- | --- |
|  |  | **T1** | **T2** | **T3** | **T4a** |
| **7th UICC edition** | **T1** | 10 (90.91%) | **1 (9.09%)** |  |  |
|  | **T2** |  | 5 (100.00%) |  |  |
|  | **T3** |  |  | 2 (100.00%) |  |
|  | **T4a** |  |  |  | 3 (100.00%) |

**Table S2** Comparison of the N classification of the 7th and 8th UICC editions depending on tumor localization

1. Floor of the mouth

|  | | **8th UICC edition** | | | | |
| --- | --- | --- | --- | --- | --- | --- |
|  |  | **N1** | **N2a** | **N2b** | **N2c** | **N3b** |
| **7th UICC edition** | **N1** | 18 (85.71%) | **3 (14.29%)** |  |  |  |
|  | **N2b** |  |  | 5 (38.46%) |  | **8 (61.54%)** |
|  | **N2c** |  |  |  | 6 (40.00%) | **9 (60.00%)** |

1. Tongue

|  | | **8th UICC edition** | | | | |
| --- | --- | --- | --- | --- | --- | --- |
|  |  | **N1** | **N2a** | **N2b** | **N2c** | **N3b** |
| **7th UICC edition** | **N1** | 9 (81.82%) | **2 (18.18%)** |  |  |  |
|  | **N2b** |  |  | 12 (75.00%) |  | **4 (25.00%)** |
|  | **N2c** |  |  |  | 2 (66.67%) | **1 (33.33%)** |

1. Lower jaw

|  | | **8th UICC edition** | | | |
| --- | --- | --- | --- | --- | --- |
|  |  | **N1** | **N2a** | **N2b** | **N3b** |
| **7th UICC edition** | **N1** | 6 (85.71%) | **1 (14.29%)** |  |  |
|  | **N2a** |  | 1 (100.00%) |  |  |
|  | **N2b** |  |  | 3 (30.00%) | **7 (70.00%)** |
|  | **N2c** |  |  |  | **3 (100.00%)** |

1. Upper jaw

|  | | **8th UICC edition** | | |
| --- | --- | --- | --- | --- |
|  |  | **N1** | **N2c** | **N3b** |
| **7th UICC edition** | **N1** | 4 (100.00%) |  |  |
|  | **N2b** |  |  | **1 (100.00%)** |
|  | **N2c** |  | 1 (16.67%) | **5 (83.33%)** |

1. Buccal plane

|  | | **8th UICC edition** | | |
| --- | --- | --- | --- | --- |
|  |  | **N1** | **N2a** | **N3b** |
| **7th UICC edition** | **N1** | 3 (60.00%) | **2 (40.00%)** |  |
|  | **N2b** |  |  | **2 (100%)** |

1. Palate

|  | | **8th UICC edition** | | | |
| --- | --- | --- | --- | --- | --- |
|  |  | **N1** | **N2a** | **N2b** | **N3b** |
| **7th UICC edition** | **N1** | 3 (100.00%) |  |  |  |
|  | **N2a** |  | 1 (100.00%) |  |  |
|  | **N2b** |  |  | 1 (50.00%) | **1 (50.00%)** |
